# Current Developments on Optical Feedback Interferometry as an All-Optical Sensor for Biomedical Applications

**DOI:** 10.3390/s16050694

**Published:** 2016-05-13

**Authors:** Julien Perchoux, Adam Quotb, Reza Atashkhooei, Francisco J. Azcona, Evelio E. Ramírez-Miquet, Olivier Bernal, Ajit Jha, Antonio Luna-Arriaga, Carlos Yanez, Jesus Caum, Thierry Bosch, Santiago Royo

**Affiliations:** 1 LAAS-CNRS, Université de Toulouse, CNRS, INP, 6 AllÃ©e Emile Monso, 31400 Toulouse, France; julien.perchoux@enseeiht.fr (J.P.); adam.quotb@enseeiht.fr (A.Q.); ermiquet@ceaden.edu.cu (E.E.R.-M.); olivier.bernal@enseeiht.fr (O.B.); antonio.lunaarriaga@enseeiht.fr (A.L.-A.); thierry.bosch@enseeiht.fr (T.B.); 2 Centre for the Development of Sensors, Instruments and Systems, Universitat Politècnica de Catalunya (UPC-CD6), Rambla Sant Nebridi 10, E08222 Terrassa, Spain; reza.atashkhooei@cd6.upc.edu (R.A.); francisco.javier.azcona@cd6.upc.edu (F.J.A.); ajit.jha@cd6.upc.edu (A.J.); carlos.yanez@cd6.upc.edu (C.Y.); caum@oo.upc.edu (J.C.); 3 Centro de Aplicaciones Tecnológicas y Desarrollo Nuclear, Calle 30, No. 502, Miramar, La Habana 11300, Cuba

**Keywords:** optical feedback interferometry, biosensors, vibrometry, flowmetry, biophotonics, metrology

## Abstract

Optical feedback interferometry (OFI) sensors are experiencing a consistent increase in their applications to biosensing due to their contactless nature, low cost and compactness, features that fit very well with current biophotonics research and market trends. The present paper is a review of the work in progress at UPC-CD6 and LAAS-CNRS related to the application of OFI to different aspects of biosensing, both *in vivo* and *ex vivo*. This work is intended to present the variety of opportunities and potential applications related to OFI that are available in the field. The activities presented are divided into two main sensing strategies: The measurement of optical path changes and the monitoring of flows, which correspond to sensing strategies linked to the reconstruction of changes of amplitude from the interferometric signal, and to classical Doppler frequency measurements, respectively. For optical path change measurements, measurements of transient pulses, usual in biosensing, together with the measurement of large displacements applied to designing palliative care instrumentation for Parkinson disease are discussed. Regarding the Doppler-based approach, progress in flow-related signal processing and applications in real-time monitoring of non-steady flows, human blood flow monitoring and OFI pressure myograph sensing will be presented. In all cases, experimental setups are discussed and results presented, showing the versatility of the technique. The described applications show the wide capabilities in biosensing of the OFI sensor, showing it as an enabler of low-cost, all-optical, high accuracy biomedical applications.

## 1. Introduction

Despite the extensive use of semiconductor lasers (SCLs) in diverse applications, from very early times, it was shown that they might show relevant instabilities when subject to external perturbations as optical reinjection, also known as optical feedback (OF). When a fraction of the radiated field from the laser is fed back into its own cavity, it induces a very rich phenomenology [1], including modulation of injection current and emitted power, changes in emission modes and even chaotic behavior, depending on the amount of energy that re-enters the cavity. Beyond the potential instabilities that may be introduced in the cavity, OF has found several applications in non-destructive testing and non-contact optical sensing through the optical feedback interferometer (OFI) (also known as self-mixing interferometer (SMI)) [2,3], where the field emitted from the laser incides upon a remote target after traveling through an optical path, so changes in the few nanometers’ scale in such an optical path between the laser and target may be monitored. The beating of the delayed optical field fed back to the cavity with that of the stationary field inside the cavity causes the optical frequency and power of the laser to change, creating observable interference fringes, with each fringe equivalent to a change of half the emission wavelength [4] in the external optical path length separating the laser and the target. Such a change contains the signature of relevant properties of changes in the optical path, which may be measured in the same laser using the built-in photodiode used as the emission monitor. The main advantage of OFI sensors is the use of the packaged laser as the light source, the interfering medium and the coherent detector all in one, making the setup extremely compact, economic, self-aligned and efficient, while keeping it contactless. Further, the resolution and accuracy obtained with the use of OFI is comparable to that of classical optical interferometry, as far as it is a coherent detection method [5].

A wide variety of applications has been approached along the years in different fields, including all types of changes in the optical path of the laser and Doppler effects in the cavity. Most of the applications include the measurement of distance changes or the velocity of the target, but also absolute distance, profiling, measurement or positioning has been approached [6,7,8]. As the signal obtained may be quite noisy, accurate signal processing and extraction of the desired parameters is required for the optimum performance of OFI sensors and has been a source of constant activity. Different approaches have been taken, including simple fringe counting (FC) [9], Fourier transform processing [10], the phase unwrap method (PUM) [11] and, more recently, the Hilbert transform [12] or wavelets [13], in order to extract the desired parameters (amplitude, velocity, distance, *etc*.) with increased accuracy. This has led to a growing number of real-world applications in sensing, as diverse as motor runout [14], integral strain [15], laser parameters [16] or acoustic fields [17], to name a few. The interest in the technique has also been accepted in different industrial environments, both in the form of patent applications or as concrete product developments [18,19].

Furthermore, although most of the activity on OFI has been traditionally happening in the short and mid-infrared wavelengths, in the last few years, an entirely full field is emerging related to terahertz self-mixing sensing, driven by the evaluation of novel quantum cascade laser designs and properties. The research activities presented are combining the simplicity and resolution capabilities of self-mixing interferometry with the particular optical properties of terahertz radiation. This is pushing forward a number of novel approaches to sensors. Applications in THz wavelengths are blooming, including large opportunities in the biomedical field [20], but also in spectroscopy [21], material analysis [22] including the detection of plastic explosives [23], and imaging, both by scanning [24] or by using a synthetic aperture approach [25].

Biophotonics and, in particular, biomedical applications are experiencing, at the same time, a trend towards better, less expensive and more compact systems, either for diagnostic or prognostic uses or in remote patient care. Such a trend is, thus, extremely well aligned with the capabilities of OFI sensors. Although the trend has accelerated in the last few years, biophotonics has been always a topic close to the developments on OFI [26], where applications in pulse shape measurements based on Doppler measurements, both in extracorporeal circulators or *in vivo* [27,28,29], have been demonstrated. Optical path change measurements on the fingernail have also enabled the measurement of the arterial pulse wave [30]. Other measurements related to body vibrations have been performed for respiratory motility [31], or ocular movements [32], or even head displacement [33]. Other approaches to the development of instrumentation have involved the combination of OFI interferometers with microscopy [34], including confocal arrangements [35].

This paper is intended to provide a general overview of the different work in progress in our labs, covering the novel developments being related to OFI techniques applied to biosensing. We have divided them into those based on sensing optical path changes and those based on Doppler sensing. Unless explicitly stated otherwise, all setups are acquiring the OFI signal from the internal photodiode of the laser, using either a USB-connected oscilloscope or an A/D conversion card connected to a PC. Only when the processing method departs from the general approach in OFI described in [2] will be explicitly described for conciseness. Along the next section, we will introduce our developments related to sensing of optical path changes, applied to the measurement of transient vibrations and to an OFI sensor for very large displacements tailored to the development of biomedical equipment for palliative care. Section 3 will outline our main developments related to flow monitoring based on the Doppler effect. We will discuss our advances in processing of the flow signals, the real-time monitoring of non-steady flows, blood flow measurements on human skin and OFI-based pressure myography. The last section will discuss the conclusions of the paper.

## 2. Optical Path Changes

### 2.1. OFI for the Monitoring of Transient Path Changes

#### 2.1.1. Introduction

The application of transient optical path changes (forced or free changes in either distance or the refractive index of the media crossed by the beam, whose statistics vary with time) is used extensively in biosensing and medical signals, usually related to vibration (e.g., in electroencephalography (EEG), electrocardiography (ECG) or vibrocardiography (VCG)) [36,37,38,39]. So far, optical feedback interferometry (OFI) has been normally used to analyze periodic vibration patterns using different signal processing schemes (outlined in the Introduction) to extract vibrational parameters, such as displacement, frequency and the average velocity of vibrating targets. While Fourier-based signal processing is an elegant approach, it requires the measurement of the complete signal over time and cannot be used in order to analyze transient signals or signals with unknown future behavior. Fringe counting, though being simple in order to process OFI signals, is not suitable when signals are time-dependent, as the threshold of detection often needs to be changed, optimized or adapted depending on the displacement of the target.

Here, we present a methodology for the analysis of transient vibrations using an OFI sensor based on an adapted processing strategy, hence broadening the scope of the measurement and making OFI sensors more suitable for their use as biosensor. To harness the power of frequency spectrum analysis without the need of a complete characterization of the signal in time, wavelet analysis provides a very suitable framework [13,40]. In such a representation, the frequency domain reflects the behavior of a temporally-localized version of the signal, thus making it suited to study a transient or undetermined signal.

#### 2.1.2. Experimental Setup

**Figure 1:**
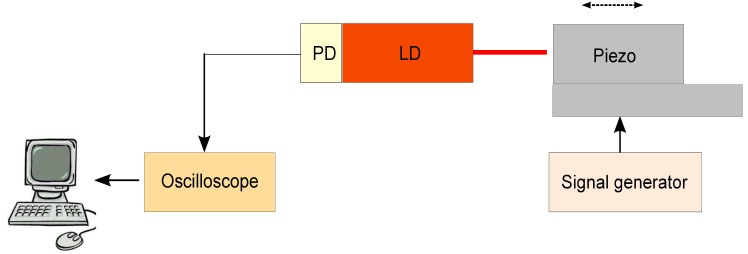
Experimental setup for the monitoring of transient path changes.

**Figure 2:**
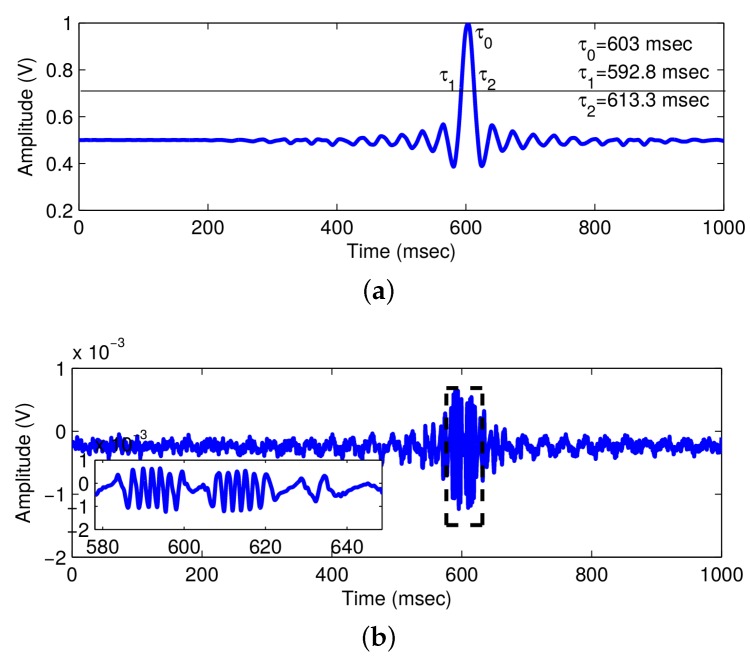
Experimental optical feedback interferometry (OFI) signal. (**a**) Transient signal applied to PZT to introduce transient vibration; (**b**) OFI signal resulting from transient motion of the piezo; the inset gives the magnified view of OFI signal produced by transient vibration.

The experimental setup is presented in Figure 1. A HL8337MG AlGaAs laser diode (LD) was used. The emission wavelength, measured with an Instrument System’s SPECTRO 320(D) R5 unit, was measured as *λ* = 692.5 nm. The optical beam emitted from the LD was focused using a Thorlabs lens 352,240 with a focal length of 8 mm and numerical aperture of 0.5 on target. The target was a piezoelectric (PZT) linear stage Phisik Instrumente-LISA (PI-LISA) (P-753.3CD), which included an embedded capacitive sensor with a resolution of 0.2 nm. The voltage to amplitude conversion factor was 3.8 µm/V. To analyze the transient response using OFI, a signal in the form of a non-periodic sinc pulse was applied to the PZT (Figure 2a). The 3-dB pulse duration (time duration for which the voltage of pulse becomes $1/\sqrt{2}$ of the maximum value) was $\tau_{3\mathrm{dB}}=\tau_{1}-\tau_{2}=613.3-592.8=20.5$ ms, where $\tau_{1}$ and $\tau_{2}$ are the first and second 3-dB points. The peak value of the pulse occurs at $\tau_{0}=603$ ms, where it presents a maximum amplitude of $V_{0}=1$ V. This perturbation in voltage induced a transient vibration of amplitude of $A_{m}=3.8$ µm =5λ/2 on the PZT. The perturbation is then measured by the OFI sensor described and the oscillation signal from the internal monitor photodiode measured in an oscilloscope connected to a computer, as in Figure 2b. It is observed that, as most of the time, the voltage signal applied to PZT is kept constant, no vibration occurs in the PZT, and no fringes are registered. At the time moment $t=\tau_{0}=603$ ms, the voltage switches to $V_{0}=1$ V, and the fringes are produced and captured (Figure 2a). The number of fringes detected $N_{f}=6$ is consistent with the amplitude of vibration [9]. The oscilloscope digitizes the analog signal from the photodiode and sends the digitized signal to a computer, where further processing is performed.

#### 2.1.3. Results

Having shown that the transient vibration of the target (PZT) is effectively detected using the OFI signal, the use of wavelets and algorithms to process the OFI signal may be directly applied (see [13,40] for a detailed description). We propose an algorithm based on the Morlet wavelet, which enables one to simultaneously obtain spectral and temporal information related to the transient vibration, including its duration and the instantaneous velocity profile introduced. Wavelet transforms are functions that are finite both in space and time, enabling one to retrieve information both on the temporal and frequency aspects of the signal through the wavelet coefficients W(a,b). For a continuous signal x(t), W(a,b) are given by:

(1) $$W\left(a,b\right)=\frac{1}{\sqrt{a}}\int_{-\infty }^{+\infty }x\left(t\right)\psi \left(\frac{t-b}{a}\right)dt$$

with W(a,b) the coefficient of wavelet transformation and *a* and *b* the dilation (scaling) and translation (shifting) parameters. The Morlet wavelet is defined in the time domain as:

(2) $$\psi \left(t\right)=\exp\left(j\omega_{o}t\right)\exp\left(\frac{-t^{2}}{2}\right)$$

where $j^{2}=-1$, and $\omega_{o}$ is set to 5.336 [41].

**Figure 3:**
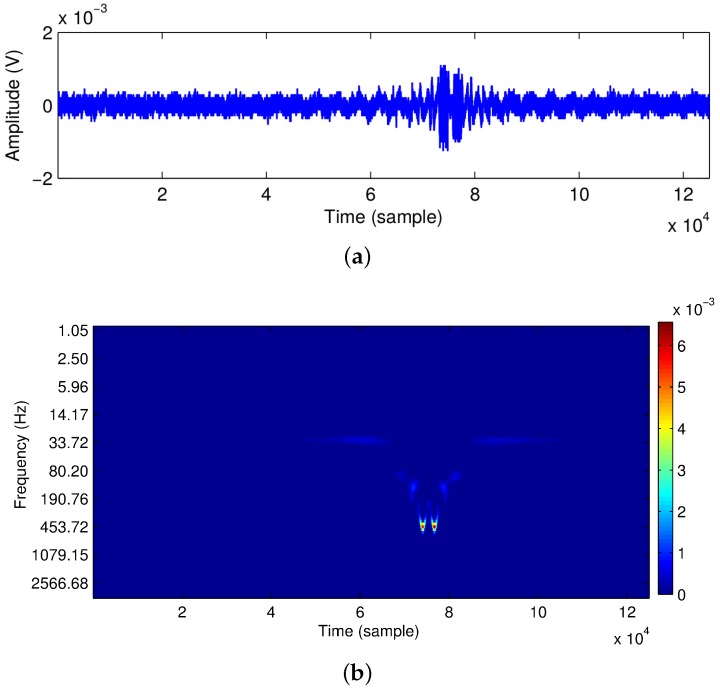
Time-Frequency representation of the OFI signal. (**a**) OFI signal; (**b**) scalogram of the OFI signal.

In our approach, a continuous wavelet transform (CWT) has been calculated directly on the signal, allowing both noise removal and detection of the relevant parameters of the signal in a single step. Such calculations are performed using a MATLAB code, typically in a few seconds, so its real-time implementation is feasible. Figure 3a shows the captured OFI signal, while Figure 3b shows the associated scalogram of the OFI signal. The scalogram represents the calculated wavelet coefficients in the time-frequency plane W(f,t), where *f* and *t* stand for frequency and time, respectively. It is observed that for most of the time of the experiment, the wavelet coefficients stay close to zero (representing the absence of a spectral component). During the pulse period, however, their values change significantly, demonstrating the rise of novel spectral components resulting from the Doppler shift along the pulse duration. From Figure 3b, the Doppler frequency is $f_{d}$ = 453.72 Hz.

**Figure 4:**
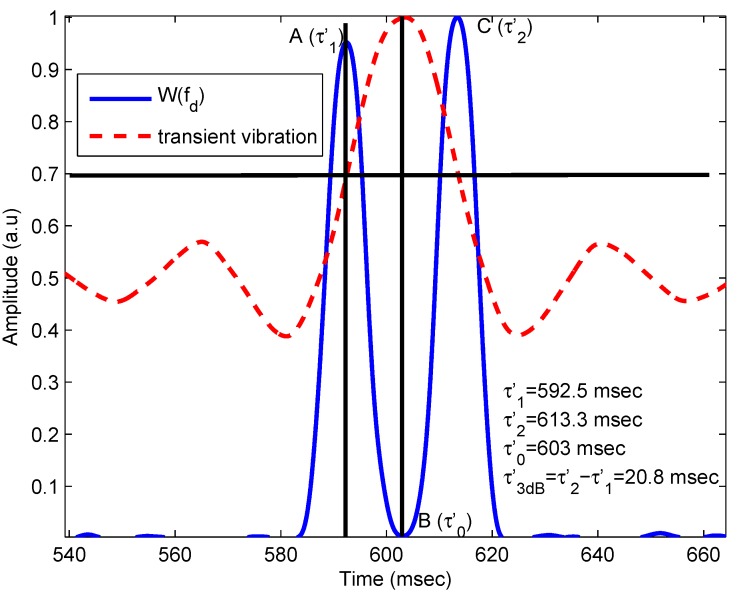
Experimental results. Characterizing the pulse: determining the 3-dB width and center time of the pulse.

To find the 3-dB pulse duration of transient vibration, the wavelet coefficients at *f* = $f_{d}$ = 453.72 Hz for all time, *i.e.*, $W(f_{d})$ are extracted from the scalogram. For simplicity, only the wavelet coefficients in the vicinity of the pulse are presented in Figure 4. It is observed that the value of $W(f_{d})$ is significant only during the pulse duration and is zero anywhere else. The plot of $W(f_{d})$ has two maxima at $\tau_{1}^{\prime }$ and $\tau_{2}^{\prime }$ with a sharp dip at $\tau_{0}^{\prime }$ represented by Points A, B and C, respectively, in Figure 4. The 3-dB width ($\tau_{3\mathrm{dB}}^{\prime }$) and center of pulse ($\tau_{0}^{\prime }$) are calculated from Figure 4 as:

(3) $$\tau_{3\mathrm{dB}}^{\prime }=\tau_{2}^{\prime }-\tau_{1}^{\prime }=613.3-592.5=20.8\;\mathrm{ms}$$

(4) $$\tau_{0}^{\prime }=603\;\mathrm{ms}$$

showing very good correspondence between the induced perturbation and the measurement extracted from the OFI signal, as may be observed in Table 1.

**Figure 5:**
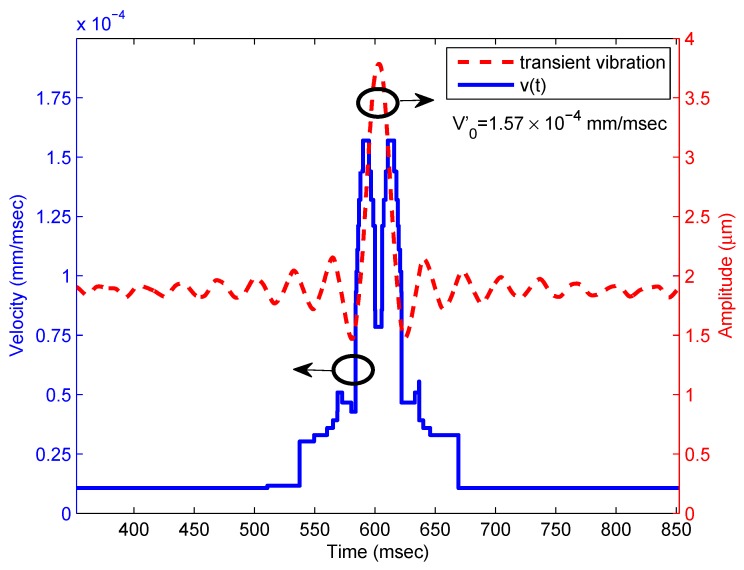
Experimental results. Velocity profile of the transient pulse determined using wavelets (blue) and time-dependent signal applied to the target (red). Circles indicate the axis for each figure.

Finally, we will determine the velocity profile v(t) of the displacement from the scalogram. First, the maximum wavelet coefficient present at each instant of time is obtained from Figure 3. Then, the corresponding frequency is found, which gives the value of the Doppler frequency shift as a function of time $f_{d}\left(t\right)$. Once the instantaneous frequency is known, the velocity of the target is obtained using $v\left(t\right)=f_{d}\left(t\right)\lambda /2$ and reconstructed in time in Figure 5. The amplitude plot of the signal used for the excitation of the piezo has been plotted to enable comparison. It is observed that the calculated peak velocity of the target is $V_{0}^{\prime }=1.57\times 10^{-4}$ mm/ms, which may be compared to that of the actual, of $V_{0}=1.65\times 10^{-4}$ mm/ms (from Figure 5, showing also that the target makes a displacement of 3.8 µm in 23 ms). In addition, a sharp dip in the velocity profile is observed consequent to the fact that the target has reached the maximum value of the perturbation and now vibrates in the opposite direction. As the signal applied to the piezo corresponds to a sinc function with time, some bias has been added to the amplitude signal to avoid negative polarization of the PZT. The oscillations around the peak are due to the shape of the sinc function and are recovered in the velocity profile. The combination of the bias and the oscillations of the sinc function result in a small velocity component due to the Doppler shift.

The method performs very accurately regarding the detection of time events, providing a convenient method for the analysis of time-dependent phenomena in OFI signals. Thus, complete and accurate characterization of transient vibrations using a Morlet wavelet approach has been demonstrated, presenting a novel, useful tool for the detection and analysis of biomedical signals, including the characterization of its time and speed signatures.

### 2.2. OFI Sensor for Large Displacements

Self-mixing has been used for displacement sensing [42], though the presence of speckle noise, which causes variations in both the amplitude of OFI signals and the value of the optical coupling factor *C*, has often reduced the range or even the possibility of correct measurement. Different approaches have been developed to cope with speckle effects, such as:

- Using two piezo-actuators to move a lens for a speckle-tracking technique [43],
- Using a voltage-controlled liquid lens and a double-headed laser diode sensor with different laser beam spot sizes have also been proposed to avoid speckle [44]
- Keeping the operating point of the OFI interferometer fixed at the half-fringe [45]
- Using OFI signal envelope tracking and dynamic fringe detection [46]
- Using the Hilbert transform and/or the wavelet transform to detect [12,13].

Only the last three methods demonstrate signal processing techniques that correctly extract and process OFI signals corrupted by speckle, while avoiding additional optical/electro-mechanical components. However, the method based on feedback techniques [45] has a limited range of operation (<100 µm) compared to the last two open loop approaches.

**Figure 6:**
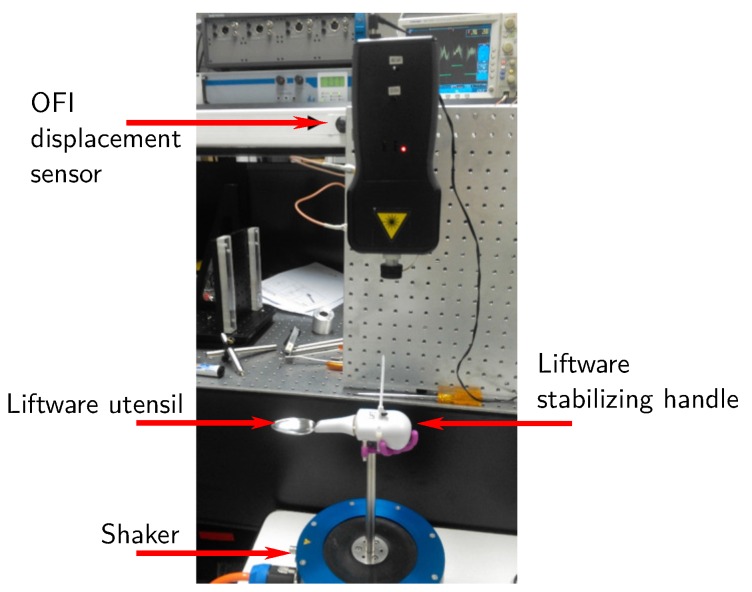
Experimental setup to measure the vibration of the stabilizing handle and the vibration of the utensil attachment.

**Figure 7:**
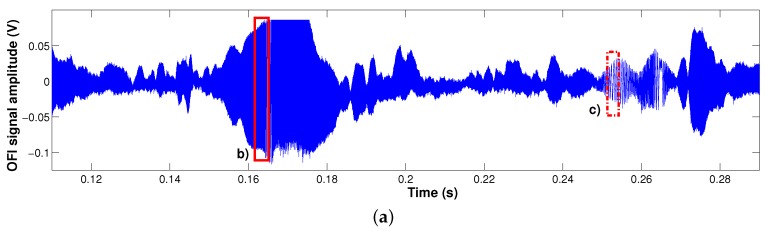

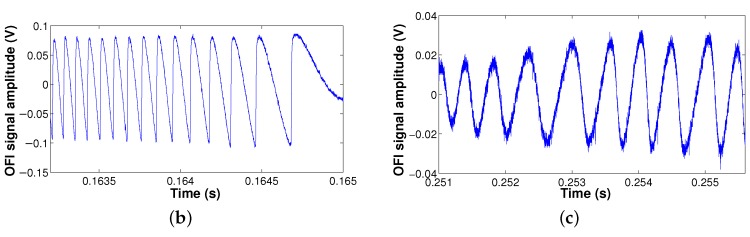
Measured OFI signal obtained using the experimental setup described in Figure 6 exhibiting strong variations in both amplitude an optical coupling factor *C*. (**a**) Full acquisition that highlights the strong amplitude modulation induced by the speckle phenomenon; (**b**) truncation of the time-domain signal showing a high *C* value; (**c**) truncation of the time-domain signal showing a lower value for the optical coupling factor *C*.

**Figure 8:**
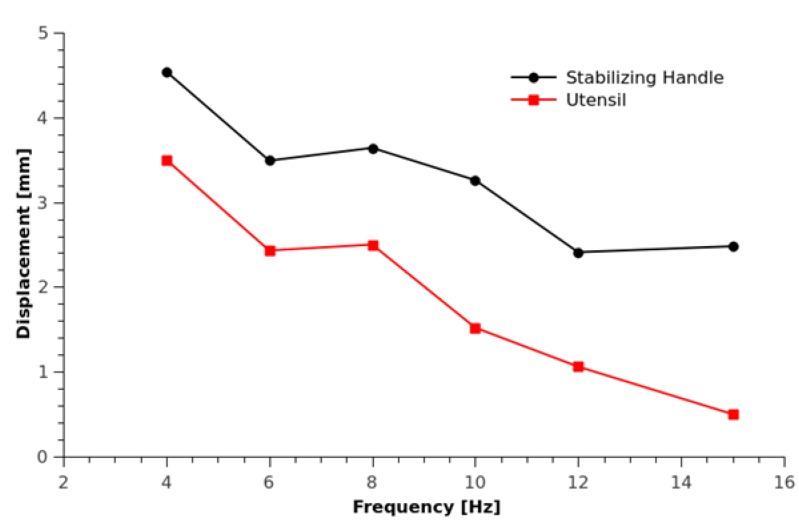
Measured vibration displacement of the stabilizing handle and the vibration of the utensil attachment induced by a shaker at different stimuli frequencies.

Here, as an example, the OFI technique has been successfully applied to quantify the displacements of the Google Liftware spoon (Figure 6), in order to determine its efficiency to damp hand-shaking vibration due to diseases, such as Parkinson. The experimental setup is shown in Figure 6. For the same given frequency and amplitude vibration, both the displacement of the handle and of the stabilized utensil are measured to estimate the Liftware system efficiency. Note also that the Liftware is placed on top of a steel post in order to reduce the parasitic influence of the magnet of the shaker. Due to the non-linearity of the induced movement and the amplitude of the displacements (a few millimeters), the OFI signals obtained with an oscilloscope from the internal photodiode are unavoidably affected by a dynamically-changing coupling factor, plus the presence of speckle, as shown in Figure 7. Consequently, in order to detect these corrupted OFI fringes, the method used here is based on the Hilbert transform. The results are summarized in Figure 8. The biggest induced displacement (4.56 mm) on the handle was obtained at 4 Hz, while the smallest (2.48 mm) at 15 Hz. Depending on the shaking frequency, it can be observed that the stabilizing handle can effectively suppress vibrations from 25% (at 4 Hz) up to 80% (at 15 Hz) of the utensil. While further work is necessary to properly characterize the Liftware’s transfer function, notably, in terms of the induced movement, it can be foreseen that the OFI configuration coupled to a robust signal processing is an adapted low-cost solution for this task.

## 3. OFI for the Monitoring of Flows

### 3.1. Processing of OFI Flow Signals under Different Scattering Regimes

#### 3.1.1. Introduction to Signal Processing for Flow Monitoring

In OFI-based flowmetry, the Doppler spectrum is analyzed to obtain the information regarding the velocity of moving particles. Since the Doppler spectrum is generated from the feedback of particles embedded in the fluid, its morphology strongly depends on the velocity distribution of particles inside the sensing volume. Thus, the number of scattering particles in the sensing volume is also an important factor in spectrum distribution. Depending on the particle concentration in the fluid, single scattering or multiple scattering may occur in the sensing point, causing a significant change in signal power spectrum of the OFI sensor. Thereby, based on the type of power spectrum morphology (*i.e.*, narrow peak, flat distribution or a slow decay), different signal processing methods have been recently proposed to accurately extract the Doppler frequency from the power spectrum corresponding to the average fluid velocity at the measurement volume. The commonly-used methods include Doppler frequency peak estimation [31], cutoff frequency approximation [47] and weighted moment estimation of the power spectrum, defined by de Mul *et al.* [48] as:

(5) $$\bar{f}=\frac{M^{1}}{M^{0}}=\frac{\int_{0}^{\infty }fp(f)df}{\int_{0}^{\infty }p(f)df}$$

where $\bar{f}$ is the average Doppler frequency (*f*), $M^{1}$ is the first moment, proportional to the average velocity times the number of particles generating Doppler shifts in the sensing volume, $M^{0}$ is the zero moment, related to the number of particles generating Doppler shifts in the sensing volume, and p(f) is the power spectrum density of the OFI signal obtained as the square module of the FFT signal.

Fluid flow profile measurement in single scattering fluids has been already presented in the literature [49]. In this section, fluid flow profilometry in both single and multiple scattering fluids is demonstrated using the cutoff frequency approximation and weighted moment estimation methods, respectively, showing the feasibility to perform measurements under the multiple scattering conditions typical of living tissue.

#### 3.1.2. Experiment

**Figure 9:**
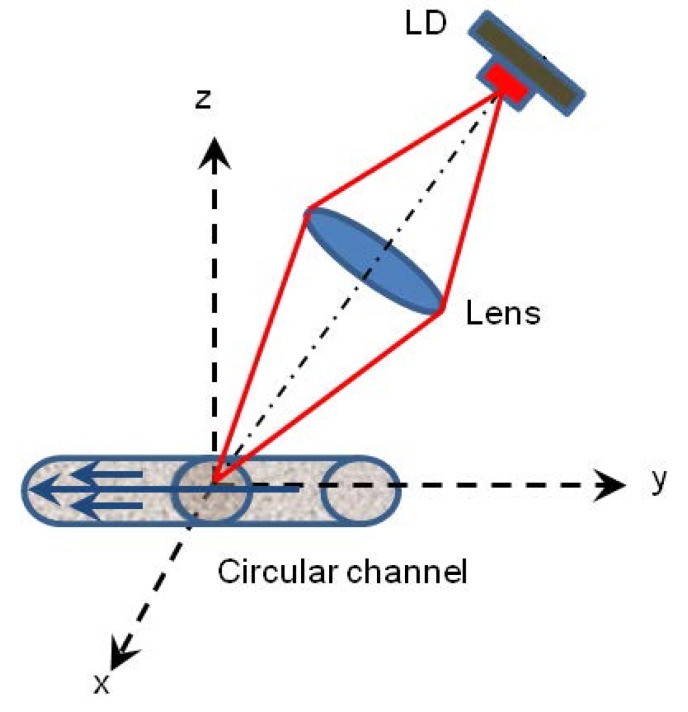
Experimental configuration for fluid flow profile measurement.

In the experimental configuration shown in Figure 9, a Thorlabs laser diode (L785P090, Newton, NJ, USA) emitting at 785 nm was used. The laser beam was focused on the circular PDMS fluidic channel with a diameter of 320 µm by an aspheric lens (C240-TME-B, Thorlabs, Newton, NJ, USA). The laser beam was set at an 80${}^{\circ }$ angle relative to the fluid flow direction, and the OFI signal from the internal photodiode was registered using a National Instruments A/D card connected to a PC.

A syringe pump (Picoplus, Harvard Apparatus) was used to pump the fluid into the channel with rate of 100 µL/min with ±0.5% accuracy. The laser and the lens assembly were mounted on a DC motorized stage (Zaber Technologies) for scanning purposes. To measure the fluid profile, the channel is scanned by the laser beam, which focused on the center of the channel using the motorized stage.

**Figure 10:**
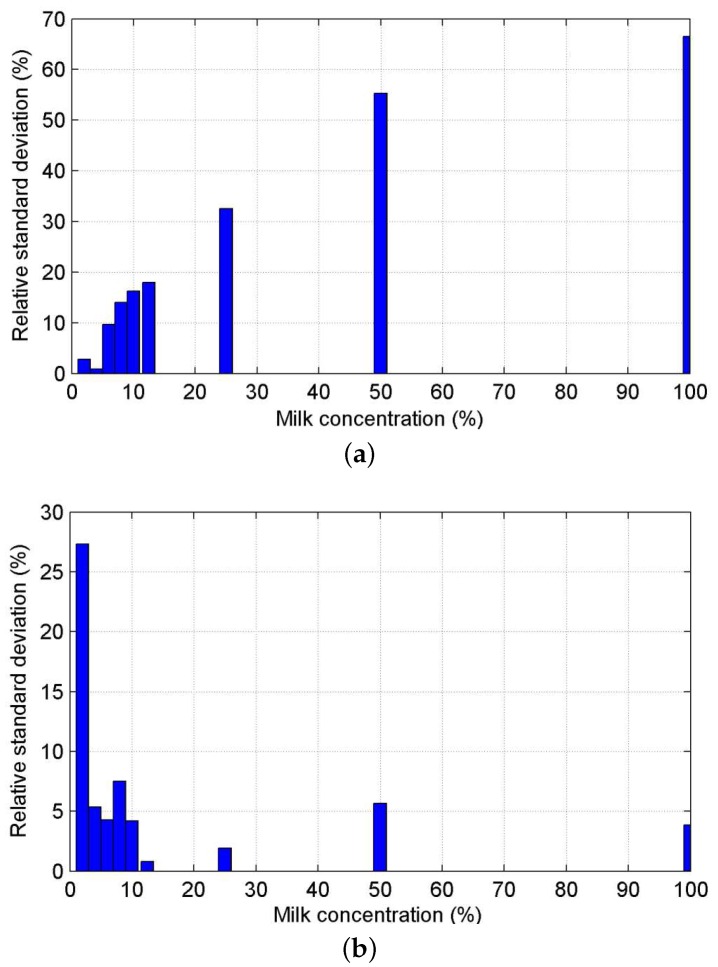
Relative standard deviation of the measured average fluid velocity profile for the circular channel (320-µm diameter) with a 50-µL/min flow rate with various dilution ratios of milk (from 2% to 100% of full milk in water) (**a**) with the cutoff frequency method and (**b**) with the weighted moment method.

To evaluate the reliability of the two existing methods with regards to the scattering regime of the fluids, different dilutions of full cream milk were used, as it is considered a good optical phantom for blood [50]. The milk dilutions in distilled water were 2%, 4%, 8%, 10%, 12.5%, 25%, 50% and 100%. The relative standard deviation (Figure 10) was computed and the results shows explicitly that for low milk concentration (single scattering), the cut-off frequency method (Figure 10a) is accurate and reliable (standard deviation of a few percent), while as soon as the multiple scattering arises (dilution ratios higher than 5%), this method is less robust. On the contrary, the weighted moment method (Figure 10b) is not reliable for very low scatterer concentrations (dilution ratio lower than 4%), while it shows accurate and stable results for high concentrations.

**Figure 11:**
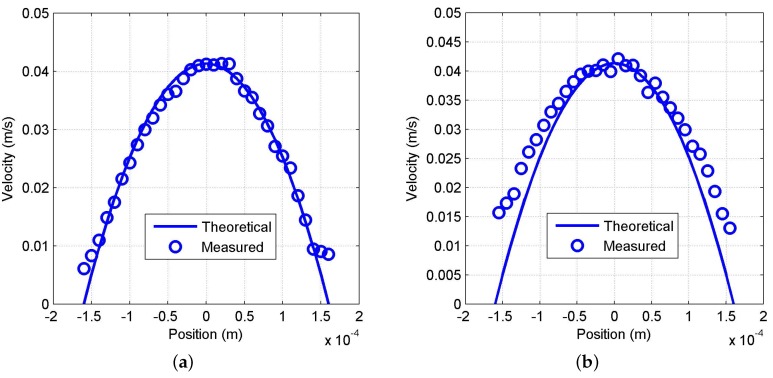
Experimental (circles) and theoretical (solid line) fluid velocity profile for the circular channel (320-µm diameter) with a 100-µL/min flow rate of diluted milk: (**a**) 2% *w*/*w* dilution, profile obtained by cutoff frequency method; (**b**) 10% *w*/*w* dilution, profile obtained by the weighted moment method.

Figure 11a,b shows the average velocity profile of the circular channel for 2% and 10% milk dilutions, respectively. The solid line in the figures shows the theoretical estimation of the velocity profile.

As seen in Figure 11a, at a 2% milk dilution, the measured flow profile using the cutoff approximation method is in good agreement with the theoretical estimation. Likely, at 10% milk dilution (Figure 11b, in the measured profile using weighted moment estimation, a good agreement can be seen in the positions close to the center of the channel. However, a significant deviation from the theoretical profile at the positions close to the channel walls is also observed. This deviation is probably due to the poor accuracy of the weighted moment estimation method at low velocities. However, OFI measurements in multiple scattering conditions in the central regions of the flow are feasible for the first time using the first-order moment approach, suggesting that further signal processing improvements may lead to complete characterization of the flow.

### 3.2. Real-Time Monitoring of Non-Steady Flows

While recent applications of optical feedback interferometry show a growing interest for flow monitoring in both the micro- and milli-fluidics domain, most of the related achievements are based on the observation of steady and unidirectional flows [49,51,52], which as commented previously have a very relevant role in biomedical signal processing. Moreover, previously-reported signal processing usually requires careful and time-consuming calculations, which is a severe limitation for further real-time implementation on embedded sensing systems. Here, our goal is to evaluate the feasibility of real-time acquisition of non-steady flows and to identify the key parameters involved.

#### 3.2.1. Methodology

The methodology proposed targets identifying the experimental challenges for real-time acquisition and processing of the flow rate of a millimeter-scale channel filled with full milk. Milk is of major interest in fluidic systems, as it is usually considered as a valid phantom for blood. The milk particles (mostly fat aggregations) are the tracers embedded in the fluid. Because of the very large concentration of particles in full milk, the sensors operates in the multiple scattering regime and, thus, the spectral analysis of the OFI signals from the internal photodiode is not a direct image of the velocity distribution of the particles that are passing through the laser beam during the oscilloscope acquisition frame.

Under such scattering conditions, the methods used by [49,53], which can be summarized as associating the maximum observed frequency in the OFI signal spectrum with the maximum velocity inside the channel, cannot be used, and some weighted moment method shall be used. For this implementation, we have used a method derived from the zero moment as:

(6) $$M=\sum_{f=0}^{f=\frac{F_{s}}{2}}\left|{\mathrm{OFI}}_{\mathrm{flow}}-{\mathrm{OFI}}_{\mathrm{no}\mathrm{flow}}\right|$$

but similar results have been found using the standard zero moment method described above. In Equation (6), the reference spectrum is obtained with *in situ* conditions, but without any flow. In order to avoid singular measurements, the reference is performed with two different levels of averaging: a first averaging consists of acquiring several spectra from a single signal acquisition, while in a second step, the first procedure is repeated three different times.

**Figure 12:**
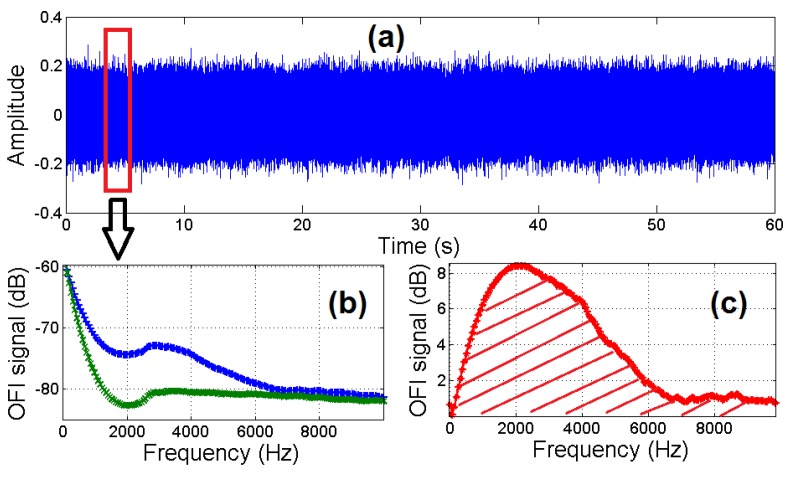
Overview of the signal processing scheme: (**a**) time domain OFI signal while fluid is pumped inside the channel; (**b**) FFT of the OFI signal for circulating fluid (blue) and with the absence of flow (green); (**c**) difference between the OFI signal for flow and the reference.

A general overview of the processing scheme is presented in Figure 12.

**Figure 13:**
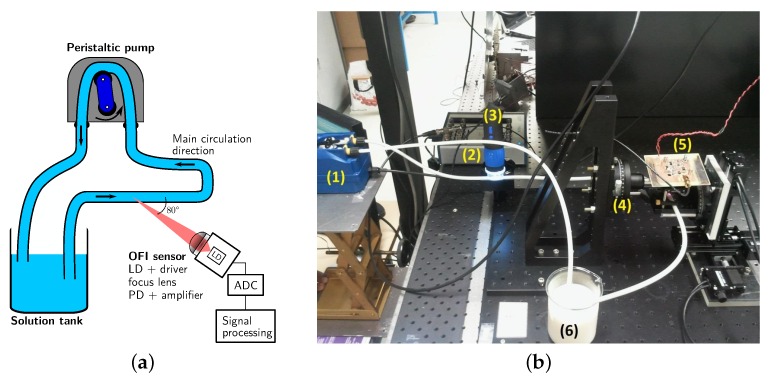
(**a**) Description of the milli-fluidic circuit with the associated OFI sensor; (**b**) photography of the experimental setup with: (1) the peristaltic pump; (2) the USB camera; (3) the NI-6361 ADC board; (4) the goniometer; (5) the OFI sensor; and (6) the solution tank.

The milk is injected by a two-squeezers peristaltic pump allowing for a manual regulation of the average flow-rate and exhibiting severe and steep changes of the instantaneous flow rate (see Figure 13). The solution is then flowing in a semi-transparent polyester tubing with internal diameter of 3.5 mm. An USB camera has been used to determine the periodicity of the pump and the distance traveled by the fluid at each cycle by tracking an air bubble circulating in the tubing.

#### 3.2.2. Characterization of the Sensor and Evaluation of the Signal Processing

**Figure 14:**
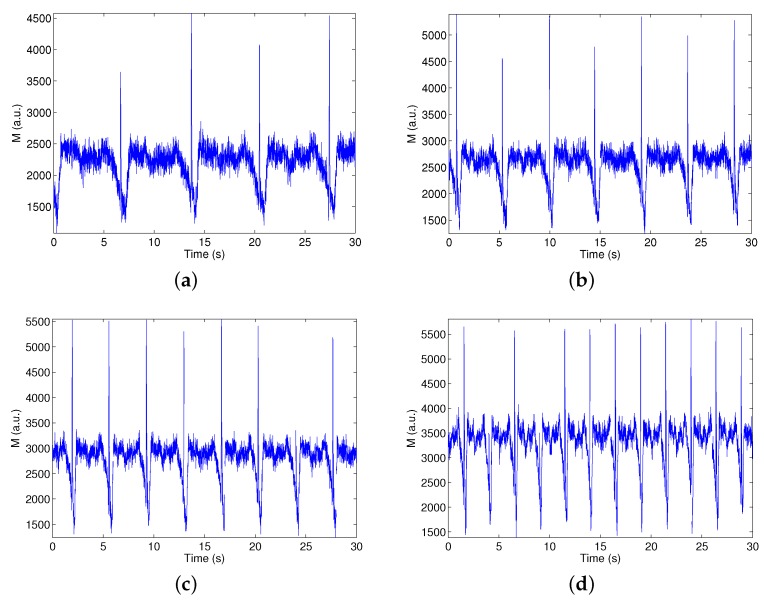
Evolution of the parameter *M* in time over 30 s for various flow rates: (**a**) potentiometer in Position 1; (**b**) potentiometer in Position 4; (**c**) potentiometer in Position 6; and (**d**) potentiometer in Position 8.

The evolution of the measured parameter *M* is presented in Figure 14 over a total period of 30 s and with different flow rates fixed by the potentiometer controlling the peristaltic pump flow (scaled from zero to eight). As can be observed, the pump cycle is explicitly visible in the plots, and the measurements are very repeatable at each cycle of the pump. Let us highlight that this algorithm allows one to observe the instants where the non-steady regime produces a rapid suction and the flow is pulled back, through the spikes that appear just before the minimal values of parameter *M*.

**Figure 15:**
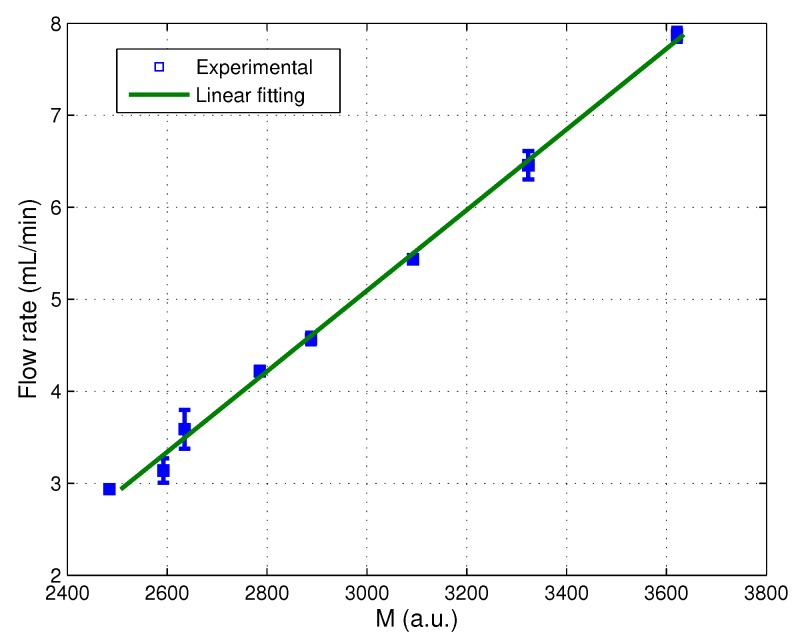
Measurement of the evolution of parameter *M* with the mean flow.

By averaging the value of the parameter *M* over 30 s, we have measured the correlation of the averaged *M* with the average flow rate (Figure 15), which in our opinion confirms the validity of the proposed signal processing. A fitting of these experimental values shows a linear regression with a slope equal to 0.004, with a determination coefficient $R^{2}$ = 0.99, which is consistent with previous reports, where this linear regression was also obtained [49,54].

#### 3.2.3. Real-Time Implementation

The real-time implementation of the algorithm has been done using MATLAB^®^ software with the data acquisition toolbox. Since the host operative system cannot guarantee determinism to plot the calculated points, we have relaxed the real-time constraint (1/dt) by averaging a larger number of FFTs and, thus, increasing the acquisition time before a new arrival of samples. Thus, the experimental imposed constraint of 8.19 ms between each measurement of *M* (for $F_{s}$ = 500 kHz and *N* = 4096 points) has been extended to 81.92 ms by averaging 10 FFTs for each point.

**Figure 16:**
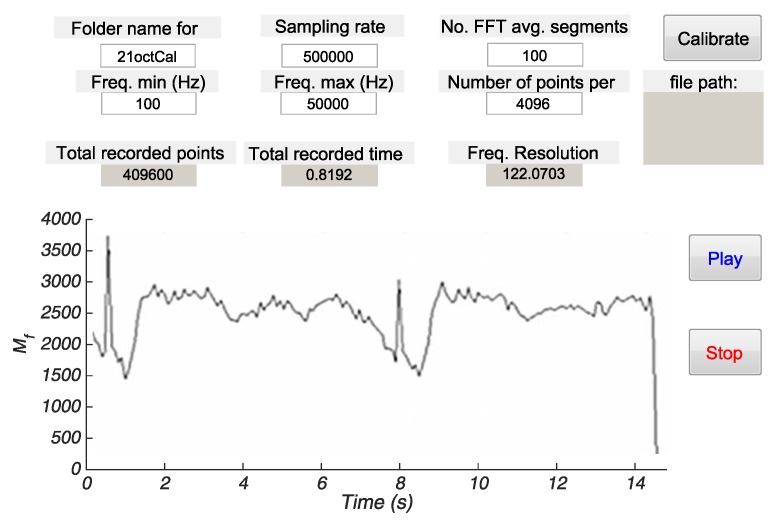
Graphic user interface with real-time monitoring of the instantaneous flow with the pump’s potentiometer at the minimal value similar to Figure 14a.

As can be appreciated in the developed front panel of the instrument (Figure 16), the pump’s period reconstruction agrees with the off-line characterization, showing the feasibility of real-time monitoring of time-dependent flows.

### 3.3. Human Skin Blood Flow Measurements

Evaluating the skin micro-capillary network is one important parameter for skin cancer diagnosis. Indeed, when a melanoma is malignant, a huge vascular network is created on the surface of the skin around the region. In that perspective, a new type of micro-capillarity skin flow sensor is presented. The main idea of this system is to demonstrate the ability to distinguish a normal skin area from a highly vascularized one.

#### 3.3.1. Skin Preparation

**Figure 17:**
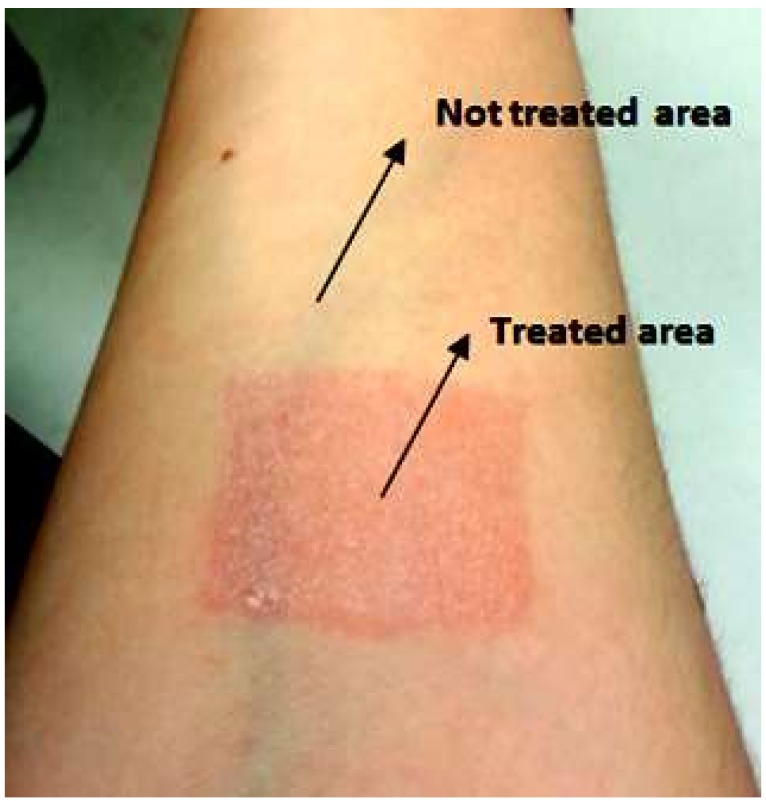
Effect of the camphor-based cream: the red region shows where the cream has been applied.

In order to validate our system, the skin of different persons is treated with a camphor-based cream (“Baume des Chochottes”). When applied on the skin, camphor causes localized vasodilatation, giving a feeling of warmth; after 10 min by the application on the skin, it is possible to observe that the skin looks more red, which indicates that the area is more vascularized than the normal skin (Figure 17).

In order to observe the evolution of the skin vascularization along time, for four persons, three different tests were performed using an OFI-based sensor, which registered the OFI signal from the internal photodiode using a National Instruments A/D card. Measurements were taken before the application of the cream, 5 min after the application and 25 min after the application. The capability of the OFI sensor to detect the increased blood flow due to the application of the cream is expected to be measurable.

#### 3.3.2. Experimental Setup

**Figure 18:**
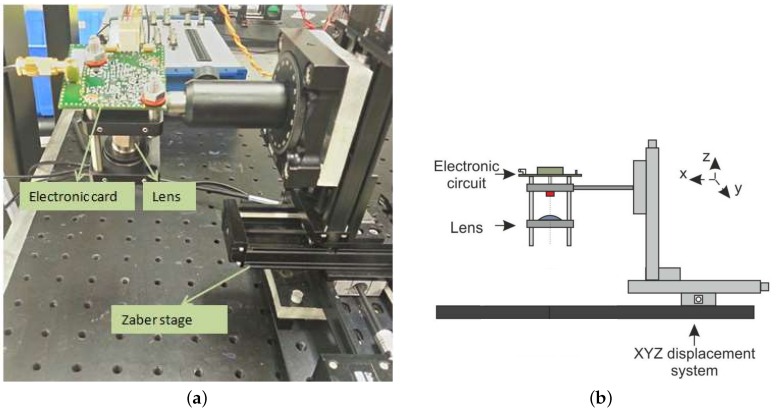
Optical setup. (**a**) Image of the optical setup with the described components; (**b**) Graphical layout of the experiment.

**Figure 19:**
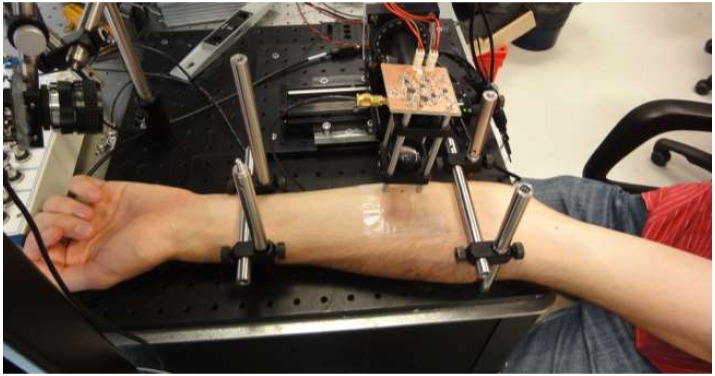
Patient position.

The setup used to conduct the experiment is shown in Figure 18 consisting of an IR laser diode with a wavelength of 1300 nm, a focusing lens and its associated electronics, consisting of the laser driver and the filtering and amplification of the detected signal, a Zaber micrometric-controlled stage for YZ displacement of the diode and some mechanical hardware accessories used to keep the arm on the table as stable as possible (see Figure 19). The coherent detection ability of the sensor has as a drawback, which is the complexity of a stable enough *in vivo* sample.

#### 3.3.3. Signal Processing

The signal processing is similar to the one presented in the previous sections and is based on the zero order moment described in Equation (6). However, while dealing with *in vivo* measurements, the OFI sensor is subject to different types of instabilities affecting the measurement conditions. In the present case, the change of the sensor-target distance (*i.e.*, the external cavity length) inherent to living systems, such as a human body, is the most important cause of signal alteration. In particular, changes in the external cavity length induce additional fringes to the ones expected from just the blood flow. Despite the mechanical effort put to limit these undesirable movements, appropriate signal processing is required.

Two differences as compared to the real-time monitoring system described in the previous section have been implemented. On the one side, the number of frequency spectrum profiles averaged for each measurement is much higher. On the other side, the signal spectrum is truncated to remove the effect of undesired fringes, since the additional fringes are usually in a much smaller frequency range than the Doppler signal, which is the target of the measurement.

Thus, the zero order moment used can be calculated as:

(7) $$M_{t}=\sum_{f=f_{\min}}^{f=\frac{F_{s}}{2}}|{\mathrm{OFI}}_{\mathrm{flow}}-{\mathrm{OFI}}_{\mathrm{no}\mathrm{flow}}|$$

${\mathrm{OFI}}_{\mathrm{flow}}$ is obtained by averaging seven FFTs acquired outside and inside the area irritated by the application of the cream. ${\mathrm{OFI}}_{\mathrm{noflow}}$ is a reference spectrum corresponding to the average of seven FFTs acquired when the laser is pointing to an immobile solid target. Then, for each area, $M_{t,\mathrm{OFI}\;\mathrm{irritated}\mathrm{skin}}$ and $M_{t,\mathrm{OFI}\;\mathrm{normal}\mathrm{skin}}$ are computed. The parameter $M_{f}$ (8) is then calculated and quantifies the variation of flow between normal and highly vascularized skins.

(8) $$M_{f}=M_{t,\mathrm{OFI}\;\mathrm{irritated}\mathrm{skin}}-M_{t,\mathrm{OFI}\;\mathrm{normal}\mathrm{skin}}$$

#### 3.3.4. Preliminary Results

Table 2 presents the moments $M_{f}$ (defined by Equation (8)) for each person measured in one point, respectively 5 and 25 min after the application of the cream.

From Table 2, we can notice that the $M_{f}$ parameter is always positive 5 min after the application, which can be explained by the vascularization enhancement that is due to the camphor. The effect of the camphor on the skin vascularization is expected to decrease with time, and in the four persons treated, the $M_{f}$ parameter measured 25 min after the application is smaller than the one measured after 5 min. We can also notice that not all of the skins behave the same way: for example, for Patient D, no major effect of the cream has been observed. Although the number of patients in this preliminary tests is still very reduced, the feasibility *in vivo* measurements of blood flow have been shown.

### 3.4. OFI Pressure Myograph Sensor

Finally, we present a pressure myograph system based on an OFI sensor. In it, a single vessel is isolated in two glass cannulas, pressurized and flushed by a liquid to simulate blood flow [55]. A scanning system supports the OFI sensor, and a signal is acquired for each position in the vessel. The OFI pressure myograph system proposed is able to measure local flow velocity with a 100-µm step and, by scanning a whole vessel section, to extract a complete flow profile.

#### 3.4.1. Aorta Cannulation

**Figure 20:**
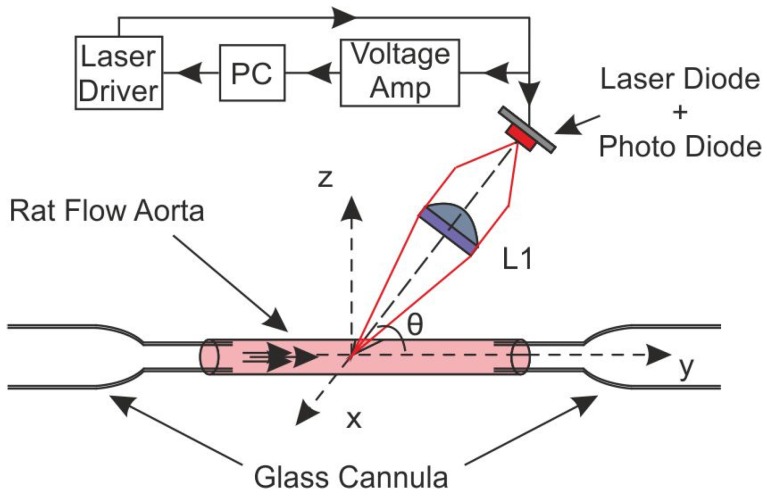
OFI pressure myograph sensor principle.

**Figure 21:**
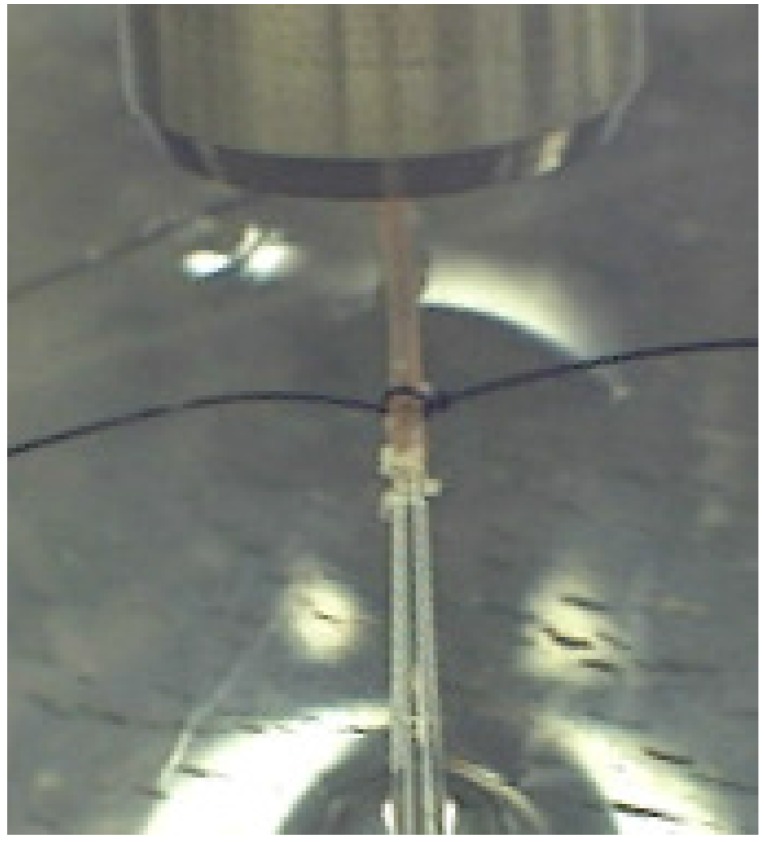
Photography of the aorta cannulation.

A frozen Wistar carotid aorta is extracted and placed on two glass cannulas. Two sutures (5-0 USP) at both extremities held the aorta in place. A high precision pressure pump (Fluigent MFCS-EZ-4C) injects a liquid composed of a ratio of 10:1 of phosphate-buffered solution and plain milk inside the aorta. We have chosen such a dilution ratio to ensure that, due to the low concentration of milk particles in the fluid, we will remain in the single scattering regime [49]. The laser sensor is focused on the center of the vessel where the Doppler frequency reaches its maximum (Figure 20). Figure 21 shows a photography of the described setup.

#### 3.4.2. OFI Sensor and Signal Processing

**Figure 22:**
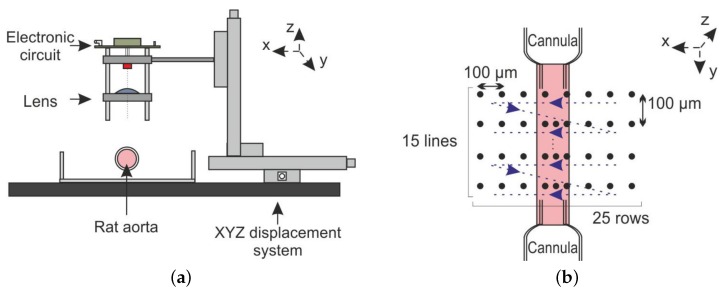
Aorta Doppler scanning system: (**a**) lateral view of the sensor scanning system; (**b**) top view of the aorta scanning process.

As depicted in the Figure 22, three miniature motorized linear stages (Zaber-LSM 50A, Zaber Technologies, Vancouver, BC, Canada), with a resolution of 0.1905 µm, compose the YZ displacement system. The amplified photodiode signal is digitized by a National Instruments card (NI USB 6251, Austin, TX, USA) connected by USB to a computer. For each measurement position, 4096 samples are recorded at a frequency of 1 MHz.

The 2D scanning protocol is presented in Figure 22a. Each acquisition is synchronized with the YZ displacement system by the computer using a LabVIEW Virtual Interface (VI). For a given position, 10 consecutive temporal signals are recorded and processed off-line using a MATLAB script/code. The fundamental Doppler frequency provides the necessary information to calculate the local velocity; thus, the kinematic properties of the flow under study can be determined. At the end of the recording process, each position datum is stored in a text file with 40,960 samples (4096 samples × 10 records). For an aorta scanning area of 3.75 mm${}^{2}$, 10 min and 160 Mb of memory are required.

The optical setup consists of a laser diode (HITACHI HL7851G) emitting at 785 nm and driven at an injection current of 50 mA. The laser is coupled to a single lens (LA1951) of a 25.4-mm focal length, and the collimated radiation is pointed to the vessel with an angle of 86°. A compact electronic system drives the laser power and the signal filtering and amplification functions.

**Figure 23:**
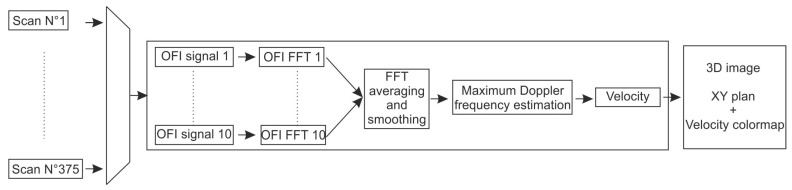
Signal processing block.

As the experiment was set to be in the single scattering regime, the signal processing consists of looking for the maximal Doppler frequency in the signal spectrum [49]. The signal processing block is presented schematically in Figure 23.

For each position, the 10 FFTs of the OFI signal are recorded, computed and smoothed. The maximum Doppler frequency is extracted by setting a threshold in amplitude of the calculated spectrum, thus associating the frequency with the maximum Doppler shift in the measurement volume.

#### 3.4.3. Photography-Based Diameter Assessment

**Figure 24:**
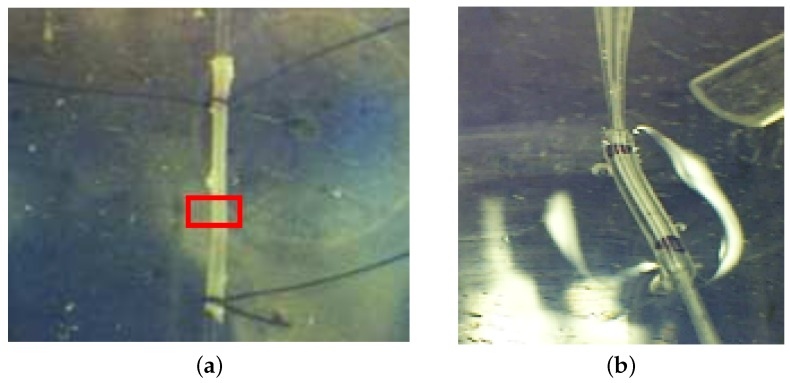
Diameter assessment: (**a**) rat aorta; (**b**) OFI calibration tube.

The internal Wistar rat aorta diameter has been evaluated to be around 850 µm [56]. Figure 24a represents the cannulated and pressurized rat aorta. Taking as a reference the 5-0 USP suture (Figure 24b), we have evaluated the pixel size at 100 µm. In that context, the internal rat aorta diameter has been evaluated as being around 800 and 900 µm, which corresponds well with the value encountered in the literature.

#### 3.4.4. OFI Diameter Assessment

In order to establish a valid measurement method, a first diameter assessment is performed on a plastic tube with a well-known diameter (Figure 24b). The sensor is calibrated using a solution of milk (1%) and distilled water (99%) flushed inside the tube at a constant velocity. Then, the beam is manually adjusted along the three axes and the angle θ where the OFI signal spectrum shows its highest frequency. The determination of the Doppler frequency spreading is realized using a threshold method. The threshold is chosen so that the detection of the spectrum cut-off is robust and weakly sensitive to the amplitude fluctuations of the signal over the complete scan of the tube. In this study, the threshold is set to 14 dB; the same value is used for the aorta measurement. When the middle of the channel is found, a scan is performed, and the velocity profile width is evaluated. Then, the result is compared to the tube diameter to evaluate the impact of the beam size in the width measurement. In a second time, the setup is kept in the same exact configuration, but the laser is beaming onto the aorta and a 2D scan performed. The fluid is flushed inside the rat aorta, and the laser beam is focused at the center of the aorta where the maximum Doppler peak is observed. As soon as the Z position is fixed, the sensor is moved to the start point of the scan, and a complete 2D scan on a surface of 3.75 mm${}^{2}$ is performed. The red rectangle in Figure 24a represents the experimental scanning area. For each scan line (along the X axis), 10 OFI signals are recorded, and an average Doppler frequency is extracted from the averaged spectrum.

**Figure 25:**
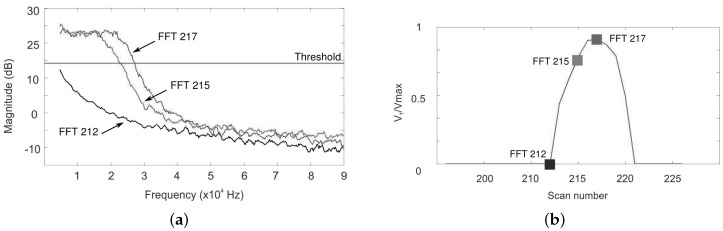
One line scan on rat aorta by OFI: (**a**) three FFT spectra: outside the aorta (FFT212), 300 µm (FFT215) and 500 µm (FFT217) inside the aorta; (**b**) Fluid velocity profile reconstructed by 25 scan points.

Figure 25 presents the results of one line scan where three points of interest have been highlighted (outside aorta (black), 300 µm (red) and 500 µm (purple) inside the aorta). The FFT spectra of these points are shown in Figure 25a and the fluid velocity profile in Figure 25b. Firstly, we can notice a difference between the spectrum outside (black curve) and inside (red and purple curves) the aorta. Secondly, it may be seen how, despite the fact that there are only 200 µm between the two positions that are situated at the vicinity of the velocity profile plateau, the maximum Doppler frequencies are well distinguished. The fluid velocity profile (Figure 25b) is composed of 25 measurements points, and from the base profile width, an aorta diameter of 850 µm is measured. Moreover, while the Poiseuille law describes the fluid velocity profile inside a tube as parabolic [57], experimental results clearly draw a parabolic shape.

**Figure 26:**
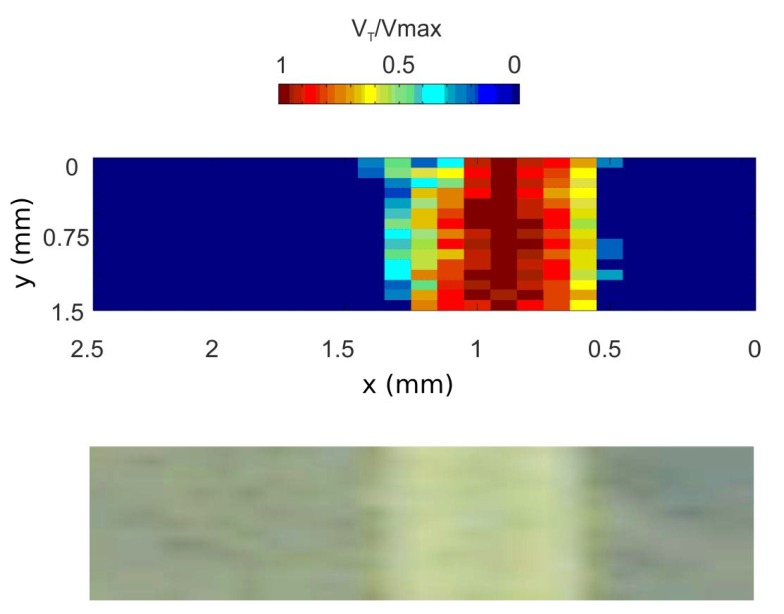
Rat aorta imaging: 2D rat aorta fluid velocity imaging captured by OFI (top image) and raw image of the scanning zone captured by camera (bottom image).

The complete aorta scan and the comparison with the raw image is presented in Figure 26. From the OFI sensor image, the aorta is clearly visible, and the fluid velocity distribution can be analyzed. High speed flow (between 0.7 and one normalized speed) is observed at 300 µm in the middle of the aorta. Near aorta walls, velocity decreases drastically due to the absence of particles. The OFI sensor can, in particular, provide with high precision the localization of aorta walls, limited mostly by spot size and scanning accuracy.

## 4. Conclusions

Interfacing OFI sensing schemes in commercial, all-purpose laser diodes together with human body measurements are a real challenge for biomedical applications, especially in *in vivo* cases, where the instabilities in the sensing experiment may be significant. However, OFI systems, as a non-invasive, compact and low-cost biosensing tool, can be a new asset for patient health monitoring and diagnosis at a wide deployment scale, following the current trend towards better and less expensive sensors for real-time health monitoring at home.

This paper gives a general overview of the OFI ability to deal with *in vivo*, *in vitro* or *ex vivo* biomaterials and signals. In that perspective, two main OFI application domains have been covered: optical path change measurements and flow measurements where OFI sensing capabilities have been applied to the biomedical field.

Optical path change schemes have been shown to cover a wide variety of applications. They may benefit from wavelet sensing to provide direct measurements of the amplitude and velocity of a sample undergoing some type of transient signal, while at the same time extracting the moment in time when the perturbation was produced. They have also been shown to cope with large displacements where speckle is the dominant source of noise.

Fluid flow measurement in biosensing using OFI techniques is mostly dedicated to monitor blood flow perfusion. Red blood cells are acting as scatterers for OFI systems. In this paper, we have demonstrated the feasibility to monitor in real time the flow rate of non-steady flow using red blood cells phantoms (milk particles) as scatter agents. This system can be directly used to monitor in real time extra-corporal blowflow, whereas further implementation would allow the system to detect bubbles inside the fluidic circuit, thus avoiding, for example, a gaseous embolism due to their presence inside the extra-corporal circuit. Furthermore, strongly concerned with flow measurements aspects, dermatology and more particularity, skin micro-capillarities can be impacted by the deployment of OFI sensing systems. The micro-capillarity network irrigates the skin and helps the body to regulate its internal temperature. In most skin cancer cases, this network is diverted in order to supply blood to the malignant tumor. To address the observation of such an effect we have developed a new tool dedicated to analyzing the micro-capillarity skin network. In order to illustrate the abilities of the OFI sensing scheme for skin cancer diagnosis, this paper demonstrates that it is possible to distinguish a highly-vascularized area (as a melanoma, for example, could induce) from the normal skin. Tests have been performed for artificial enhancement of the skin vascularization using a vasodilatation cream. From these early experiments, a new demonstrator is developed in order to fit with skin melanoma detection clinical procedure. The first clinical results are quite promising and will be published later. The last part of this paper shows that the OFI system can be used as well for *ex vivo* experiments. We show that an OFI sensor is able to reconstruct the flow profile inside a vessel in order to test and verify the mechanical functionality of tissue samples. A pressure myograph OFI system is proposed to monitor in real time the vessel diameter. Further incrementation of OFI systems for flow monitoring will address 2D and 3D imaging of complex flow networks.

## Figures and Tables

**Table 1:** Comparison between the reference and measured pulse parameters.

Parameters	Original Value	Calculated Value	Error
Center pulse time	$\tau_{0}=603$ ms	$\tau_{0}^{\prime }=603$ ms	0.0%
First 3-dB time	$\tau_{1}=592.8$ ms	$\tau_{1}^{\prime }=592.5$ ms	0.05%
Second 3-dB time	$\tau_{2}=613.3$ ms	$\tau^{\prime }=613.3$ ms	0.0%
3-dB pulse duration	$\tau_{3\mathrm{dB}}=\tau_{2}-\tau_{1}=20.5$ ms	$\tau_{3\mathrm{dB}}^{\prime }=\tau_{2}^{\prime }-\tau_{1}^{\prime }=20.8$ ms	1.4%
Peak velocity	$V_{0}=1.65\times 10^{-4}$ mm/ms	$V_{0}^{\prime }=1.57\times 10^{-4}$ mm/ms	4.8%

**Table 2:** Measured $M_{f}$ for the 4 persons tested.

	$M_{f}$ after 5 min	$M_{f}$ after 25 min
Patient A	186	154
Patient B	165	77
Patient C	89	71
Patient D	53	−36
